# Increasing the Thermal Stability and High-Temperature Strength of Vanadium Alloys by Strengthening with Nanosized Non-Metallic Particles

**DOI:** 10.3390/ma16062430

**Published:** 2023-03-18

**Authors:** Ivan A. Ditenberg, Ivan V. Smirnov, Konstantin V. Grinyaev, Alexander N. Tyumentsev, Vyacheslav M. Chernov, Mikhail M. Potapenko, Sergei A. Kulinich

**Affiliations:** 1 Institute of Strength Physics and Materials Science, The Siberian Branch of the Russian Academy of Sciences, Tomsk 634055, Russia; 2 JSC Bochvar High-Technology Research Institute for Inorganic Materials, Moscow 123098, Russia; 3 Research Institute of Science and Technology, Tokai University, Hiratsuka 259-1292, Kanagawa, Japan

**Keywords:** vanadium alloys, internal oxidation, dispersion strengthening, microstructure, microhardness, thermal stability

## Abstract

Using the methods of scanning and transmission electron microscopy, the features of the structural-phase state of a vanadium alloy of the V–Cr–Ta–Zr system after a combined treatment, which consisted in cyclic alternation of thermomechanical and chemical-heat treatments, were studied. The values of yield strength and ductility of the V–Cr–Ta–Zr alloy were determined, depending on the stabilization and test temperatures. It was established that, after the combined treatment, the structural-phase state of the V–Cr–Ta–Zr alloy was composite, in which the joint implementation of dispersion and substructural strengthening ensured the formation of a gradient grain structure with a polygonal state, the elements of which were fixed by nanosized ZrO_2_ particles characterized by a high thermal stability. Such modification of the microstructure was accompanied by an increase in the high-temperature strength and a shift in the upper limit of the temperature stability interval towards high temperatures, of up to 900 °C. It was assumed that the polygonal state inside the grains contributed to the implementation of cooperative mechanisms of the dislocation–disclination type, which ensured the accommodation of the material in the “high-strength state” under loading.

## 1. Introduction

Low-activation vanadium alloys are considered promising structural materials for a new generation of nuclear fusion reactors [1,2,3,4,5,6,7,8,9,10,11,12,13,14,15]. A consequence of the high chemical activity of refractory components with interstitial impurities (C, N, O) is the formation of solid solutions and particles of second phases [16], which transform these alloys into the category of heterophase materials. In such composite materials, the presence of nanosized non-metallic inclusions (carbides, oxides, oxycarbonitrides, etc.) in the metal matrix ensures the implementation of dispersion strengthening through the Orowan mechanism [17].

One of the main requirements of vanadium alloys as structural materials is the combination of high-temperature strength and low-temperature ductility. In accordance with modern physical concepts, the main mechanisms of strengthening of heterophase alloys at low temperatures are deformation, solid solution, and dispersion [17,18,19,20,21,22,23,24,25]. In turn, the provision of high-temperature strength (at T ≥ 0.6 T_melt_) is realized through dispersion strengthening, the improvement of the efficiency of which requires optimization of the elemental and phase composition. In this case, the determining factor is the thermal stability of the nanosized particles of nonmetallic phases. With the joint implementation of dispersion plus substructural strengthening, such particles not only pin the dislocation substructure, but also effectively fix the boundaries of grains and subgrains, which leads to the suppression of recrystallization processes [24,25].

Research in this direction is mainly focused on vanadium alloys of the V–Cr–Ti system, which demonstrate acceptable strength properties only up to 700–800 °C [10,12,26,27,28,29,30]. The use of thermomechanical treatments (TMT) promotes the dispersion and transformation of metastable carbides based on Ti and V into stable TiC, which leads to a significant improvement in strength properties at these temperatures. Unfortunately, this approach does not allow shifting the boundary of the thermal stability of the microstructure and properties to higher temperatures. The main reason for this limitation is the low solubility of carbon in vanadium [16], which limits the volume fraction of precipitated second phase nanoparticles. In addition, the thermal stability of dispersion-strengthened alloys is determined by the concentration of interstitial impurities in the solid solution.

One of the ways to increase the volume fraction of the second phase in an alloy is to use a chemical-heat treatment (CHT) by introducing oxygen, followed by segregation of oxide-based particles. As is known [16], oxygen has a good affinity for vanadium and dissolves in it at higher concentrations than carbon. In the case of the V–Cr–Ti system, this approach makes it possible to significantly increase the strength properties up to 800 °C [31].

The authors of the works in [25,32] proposed vanadium alloys of the V–Me(Cr, W)–Zr–C system with purposeful alloying with interstitial (C, N, O) and substitutional (W, Zr) elements. The main phase-forming element of the alloys of such a system is Zr, which is characterized by a high chemical activity towards interstitial impurities (C, N, O). It was found that when carbide strengthening was implemented in such systems, the strength properties at high temperatures increased by several tens of percent. In the case of using CHT, in addition to a large increase in the high-temperature strength, the thermal stability of the alloys increased by 100–300 degrees [24,25].

Thus, issues related to both the development of new approaches and the adaptation of existing TMT and CHT methods for the vanadium alloys of various systems remain topical. At the same time, the various materials science tasks include the study of the features of the transformation of the structural-phase state and the accompanying change in the complex of physical and mechanical properties at various stages of treatment.

In the report in [33], using the example of an alloy of the V–Cr–Zr system, the high efficiency of the combined use of TMT and CHT was demonstrated, for increasing the high-temperature strength, while maintaining an acceptable level of ductility.

In this work, we studied the effect of the stabilization annealing temperature on the features of the structural-phase state and the level of mechanical properties of a V–Cr–Ta–Zr system alloy after a combined treatment that consisted of alternating TMT and CHT cycles.

## 2. Materials and Methods

Vanadium alloy V–6.99 Cr–1.8 Ta–0.45 Zr–0.138 C–0.174 O–0.034 N (at. %) (hereinafter V–Cr–Ta–Zr), obtained at JSC Bochvar High-Technology Research Institute for Inorganic Materials (Moscow), was used.

A combined treatment was carried out that included three cycles of alternating TMT and CHT.

Before the first stage of TMT, a homogenizing one-hour annealing was carried out in vacuum at a temperature of 1400 °C.

The stage of thermomechanical treatment according to mode II (TMT-II) [29] provides nanostructuring of the initial heterophase structure, through implementation of the mechanism of phase transformations, by dissolving metastable vanadium carbides, followed by segregation of the stable phase from the solid solution. In this case, TMT-II included four cycles of “rolling at room temperature with deformation ε = 20% + annealing at 600–700 °C”.

CHT with the method of nonequilibrium internal oxidation, similarly to the works in [25,32,33], consisted of two stages: (1) formation of a surface scale during annealing in air at 650 °C for 5 to 30 min; and (2) vacuum annealing with a total duration of 9 h, with a stepwise increase in temperature from 620 to 1000 °C, to transfer oxygen from the surface scale into the sample.

Before mechanical tests, the samples were subjected to vacuum stabilization annealing for 1 h at temperatures (T_s_) of 1000 °C and 1100 °C.

The oxygen concentration was determined on the basis of the mass of the samples before and after CHT, through accurate (no worse than 10^–4^ g) weighing on an A&DCOLTD GH-200 electronic laboratory balance. The concentration of introduced oxygen was ≈ 0.825 at. %, and its total concentration was (C_O_) ≈ 1 at. %. The actual oxygen concentration (C_O_) was selected in such a way as, not only to bind all Zr into ZrO_2_ oxide, for which the C_Zr_:C_O_ ratio was 1:2, but also to leave part of the oxygen in the solid solution.

The obtaining and analysis of electron back scattering diffraction (EBSD) patterns was carried out using a Thermo Fisher Apreo 2 S (20 kV) scanning electron microscope equipped with an EDAX Velocity Super backscattered electron detection system. Grain structure orientation maps were obtained in the mode with a hexagonal grid. Kikuchi patterns formed by backscattered electrons were automatically indicated by the EDAX APEX EBSD software. The received data array was processed using EDAX OIM analysis software. Samples for EBSD analysis were subjected to mechanical grinding with successive reductions of the abrasive size and subsequent electrolytic polishing in 80% H_2_SO_4_ + 20% CH_3_OH electrolyte.

Transmission electron microscopy (TEM) studies were carried out using a JEOL 2100 (200 kV) electron microscope. Thin foils were obtained by double-sided sputtering with argon ions on a “Model 1051 TEM Mill” ion milling and polishing system at an accelerating voltage of 5 kV.

During the quantitative certification of the grain, defect, and heterophase structures, classical analysis methods were used (electron diffraction analysis of the phase composition, gb analysis of the dislocation structure, determination of the dislocation density, analysis of the misorientation of neighboring grains to separate low- and high-angle boundaries, analysis of special-type boundaries, and two-trace analysis of grain boundaries) [34], as well as the method of dark-field analysis of misorientations of discrete and continuous type [35], which was carried out by studying the behavior of the extinction contours when the sample was tilted in a goniometer. When identifying the structural type and determining the lattice parameters of the second phases particles, the PDF-4 database (The Powder Diffraction File 4) was used.

Mechanical tests of dog-bone shaped specimens with the gauge dimensions 13 mm × 2 mm × 1 mm were carried out with tension in vacuum ~ 3 × 10^−3^ Pa at a rate of 2 × 10^−3^ s^−1^ at temperatures of (T_t_) 20, 800, and 900 °C.

The fractographs of the samples after tensioning were studied using a Tescan Vega 3 SBH scanning electron microscope (30 kV).

Microhardness (Hit) was determined using the Oliver–Pharr method on a CSM Instruments TTX-NHT2 instrument (Berkovich tip) at various distances from the oxidation surface. The indenter loading and unloading rate was 0.5 H/min. Dwell time under maximum load (0.25 N) was 15 s. At least five measurements were carried out, to obtain average Hit values at each distance from the oxidation surface (L).

## 3. Results

Features of the structural-phase states and strength properties of V–Cr–Ta–Zr alloy, depending on the modes of TMT and subsequent annealing at different temperatures, were studied in detail in [36].

**Figure 1:**
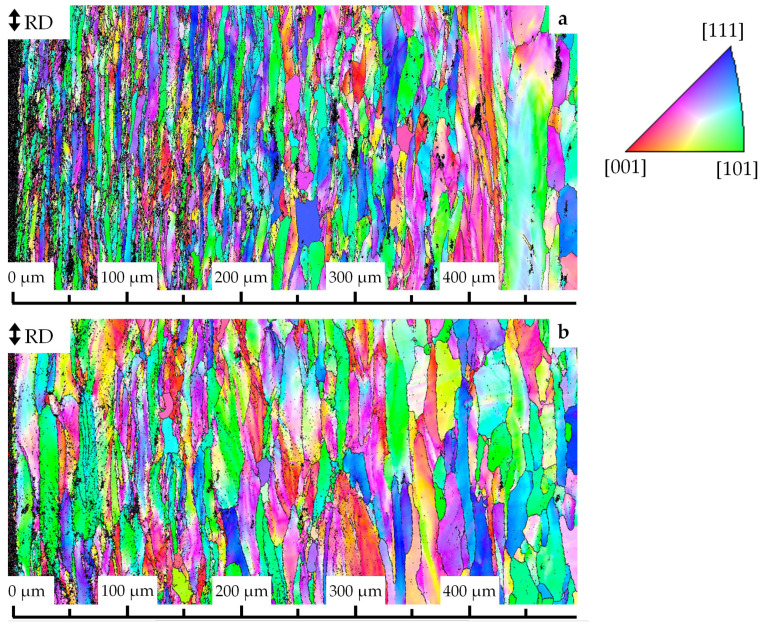
Grain orientation maps of the V-Cr-Ta-Zr alloy after combined treatment with stabilization annealing at 1000 °C (**a**) and 1100 °C (**b**). SEM/EBSD.

After the combined treatment and stabilization annealing at 1000 °C, a gradient structural state was formed in the material (Figure 1a). As can be seen, the grains were elongated in the rolling direction (RD) and their length decreased, while their width increased the farther from the oxidation surface they are located. Near the surface, the grains were 5 to 10 µm wide, while they were several hundred µm long. At a distance of 200 μm from the surface, the width of the grains varied from 10 to 15 μm, and their length was 50–150 μm. At a distance of 250–300 µm from the surface, the grain width could reach 25–30 µm. Their length, as a rule, did not exceed 100 μm.

Near the central part of the sample (400–500 μm from the oxidation surface), the structural state was identical to that observed immediately after TMT-II without stabilization annealing or annealing at temperatures below 900 °C [36]. Large grains with a length of several hundred µm and a width of 50 µm or more alternated with clusters of grains 10–25 µm wide, the length of which, as a rule, was 50–100 µm. Thus, there was a tendency for a decrease in the non-equiaxiality, which reflected the ratio of the grain’s width to their length. It is important to note that, regardless of the distance from the surface and the grain size, a gradient color was observed inside them, which indicated the presence of continuous misorientations [33].

Despite a partial relaxation in the case of stabilization annealing at 1100 °C, the structural state of the material remained gradient (Figure 1b). There was still a significant difference in the shape and size of grains in the near-surface region and at different distances from it. In this case, the grain orientation maps indicated that the relaxation processes proceed more intensively near the surface at a distance of up to 150 μm. The grain width in this region increased, compared to annealing at 1000 °C (Figure 1a), and it was from 10 to 30 µm, while the grain length was 150–200 µm. At distances from the surface of ~175 μm or more, grains 25–30 μm wide and about 150 μm long were predominantly observed. In the central part of the sample (400 μm or more), the grain width could reach 40 μm, and their length, as a rule, did not exceed 100 μm. As in the case of annealing at 1000 °C, after stabilization at 1100 °C, the gradient color was retained in all grains.

**Figure 2:**
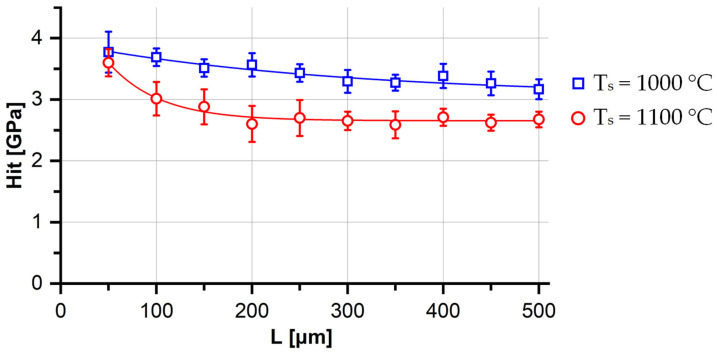
Microhardness values (Hit) as a function of distance from the oxidation surface (L) of V–Cr–Ta–Zr alloy after combined treatment with different stabilizing annealing temperatures (Ts).

The gradient structural states presented in Figure 1, were characterized by a specific change in the microhardness values with the increase in distance from the oxidation surface. In the case of stabilization annealing at 1000 °C, the Hit decreased almost linearly, from 3.77 GPa at a distance of 50 μm, to 3.17 GPa at a distance of 500 μm (Figure 2). In this case, with the exception of the near-surface region (50 μm), the spread of values (ΔHit) did not exceed 5%.

After annealing at 1100 °C, a sharp decrease in Hit values from 3.60 GPa to 2.67 GPa was observed at distances from the surface in the range from 50 μm to 200 μm. With greater distance from the surface, the average values of microhardness practically did not change. The more than 10% scatter of the Hit values in the range from 50 μm to 250 μm was a consequence of the inhomogeneous structural state, due to the high inequigranularity.

Thus, for the presented gradient states, the average grain size and average microhardness values could not be used as universal characteristics.

**Figure 3:**
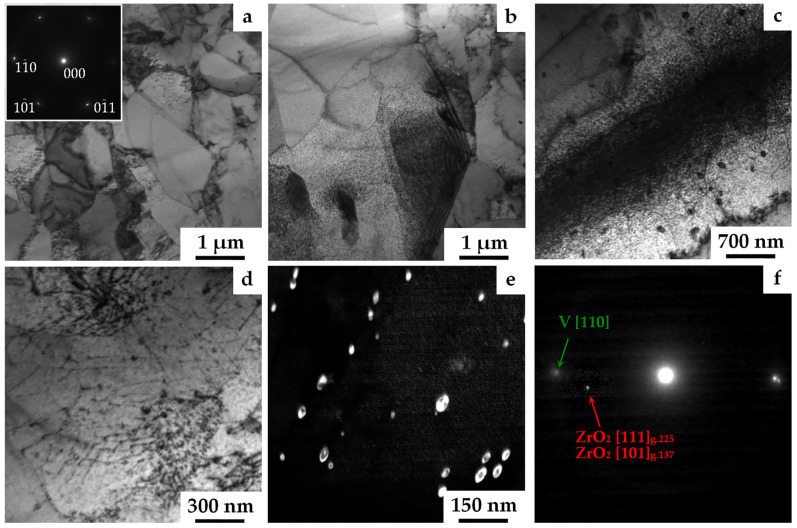
Features of the grain–subgrain, defect, and heterophase structure of the V–Cr–Ta–Zr alloy after combined treatment with stabilizing annealing at 1100 °C. (**a**) Bright-field image with the corresponding microdiffraction pattern; (**b**–**d**) bright-field images; (**e**) dark-field image; (**f**) microdiffraction pattern with matrix reflections and a coinciding reflection of the second phase; TEM.

Using transmission electron microscopy, it was found that the grains detected by EBSD analysis (Figure 1) were fragmented by low-angle misorientation boundaries into subgrains of micron and submicron sizes (Figure 3a). The corresponding microdiffraction pattern, obtained from an area of the order of several square microns, was characterized by a configuration of reflections belonging to the same zone axis. These reflections were blurred in the azimuthal direction up to 5°. These facts indicated that the presented boundaries were predominantly low-angle misorientation boundaries. Inside these blocks, a high density of narrow extinction contours was observed, which quickly moved when the sample was tilted in the goniometer. This indicated the absence of structural states with a high curvature of the crystal lattice [35]. In addition, the contrast at many boundaries was characterized by the presence of thickness extinction contours (Figure 3b), which indicated their equilibrium state. An important feature of the heterophase structure was the almost uniform distribution of high-dispersed particles (from 3 to 20 nm) at the micron scale level (Figure 3b,c). Such particles effectively pin both individual dislocations distributed inside the grains in a chaotic manner, as well as the dislocation networks and walls (Figure 3b–d). In particular, in the bright-field image (Figure 3d), dislocation networks decorated with nanosized particles were observed inside the grains. The scalar dislocation density determined using the secant method [34] was (5–9)∙10^9^ cm^−2^. In addition to very small particles, there were particles ranging in size from several tens to hundreds of nanometers (Figure 3c,d). The dark-field image obtained in the coinciding reflection from the tetragonal [101] and face-centered [111] lattices (Figure 3e) showed large and small nanoparticles with a rounded shape.

A comparison of the scanning and transmission electron microscopy data showed that a gradual change in the oxygen concentration with distance from the oxidation surface also resulted in a change in the local volume fraction of the second phase. At the same time, no predominance of particles of any size fraction with the increase in distance from the oxidation surface was found.

All particles of the second phase, depending on their size, are various modifications of the ZrO_2_ type oxide, with similar stoichiometric ratios (C_Zr_:C_O_) (Table 1). It was established that, similarly to the internally oxidized V–Cr–W–Zr and V–Cr–Zr alloys [25,33], fine particles with sizes from 3 to 20 nm were the FCC (Fm–3m) modification of ZrO_2_ with a lattice parameter a = 5.1–5.15 Å. Larger particles, several tens of nm in size, had a tetragonal modification of the crystal lattice, and their parameters varied in the ranges a = 3.49–3.67 Å; c = 4.95–5.32 Å. In addition, particles with sizes of several hundred nm or more showed a monoclinic (P21/c) modification of the ZrO_2_ lattice, the parameters of which varied in the ranges: a = 5.15–5.31 Å; b = 5.21–5.27 Å; c = 5.15–5.38 Å; β = 99.22–99.46°. The scattering of the lattice parameters near the tabular data was a consequence of deviations from the stoichiometric composition and distortions in the fields of local internal stresses.

The presented results show that only the particles of high-temperature FCC modification of ZrO_2_ were characterized by high dispersion and ensured the strengthening through the Orowan mechanism [17]. It was noted in [25] that the thermodynamic stability of such particles was associated with a change in the boundaries of the phases existence, as a result of an increase in the relative contribution of the surface energy to the free energy. The advantage of changing the type of the crystal lattice (sequence: monoclinic → tetragonal → FCC) with the decrease in the size of ZrO_2_ particles is associated with a significant effect of the surface energy on the phase transition temperature [37].

**Figure 4:**
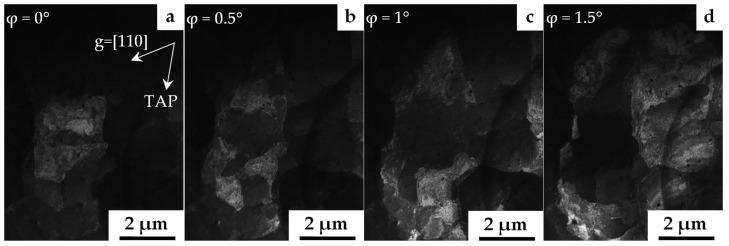
Polygonal structure of the V–Cr–Ta–Zr alloy after combined treatment with stabilizing annealing at 1100 °C. Dark-field images in the [110] reflection at different tilt angles in the goniometer (ϕ). (**a**) ϕ = 0°; (**b**) ϕ = 0.5°; (**c**) ϕ = 1°; (**d**) ϕ = 1.5°. TAP is the projection of the goniometer tilt axis; TEM.

Figure 4 shows an example of a dark-field analysis of misorientations in the V–Cr–Ta–Zr alloy after combined treatment with stabilizing annealing at 1100 °C.

The analysis of the behavior of the extinction contours made it possible to establish that a polygonal structural state was formed inside the grains. In this case, low-angle misorientation boundaries, which, among other things, are dislocation networks and walls, are characterized by a misorientation vector from tenths of a degree to several degrees. The sizes of the polygons, separated by low-angle boundaries, ranged from a few tenths of a micron to several microns. The curvature of the crystal lattice inside the presented polygons, characterizing the continuous nature of the change in crystal orientation, as a rule, did not exceed a few deg/μm. Using a detailed dark-field analysis of the discrete misorientations, no twin boundaries or special boundaries were found.

**Figure 5:**
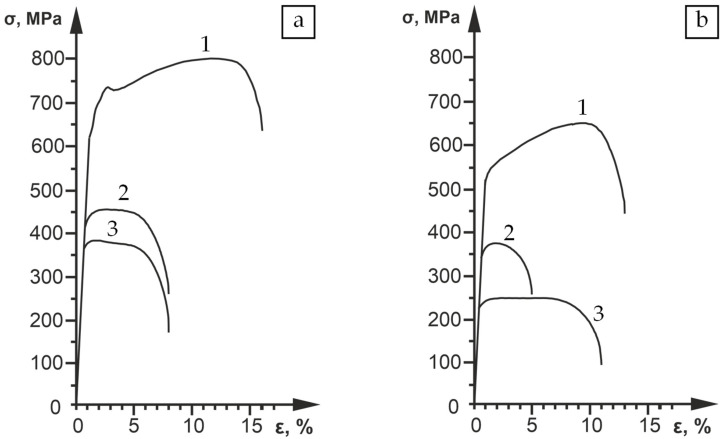
Tensile (σ-ε) curves at different test temperatures (T_t_) (1—T_t_ = 20 °C; 2—T_t_ = 800 °C; 3—T_t_ = 900 °C) of V–Cr–Ta–Zr alloy samples after combined treatment. (**a**) Stabilizing annealing temperature = 1000 °C; (**b**) stabilizing annealing temperature = 1100 °C.

Figure 5 shows the tensile (σ-ε) curves at 20, 800, and 900 °C for the samples of V–Cr–Ta–Zr alloys after combined treatment with different stabilizing annealing temperatures. Table 2 shows the corresponding values of yield strength (σ_0.1_) and ductility (δ).

The use of a combined treatment led to a significant increase in the values of the yield strength compared to TMT-II [36]. After TMT-II, carbide strengthening of this alloy provided values σ_0.1_ = 315 MPa and δ = 29% at 20 °C and σ_0.1_ = 185 MPa and δ = 29% at 800 °C. In the case of combined treatment with stabilization at 1100 °C, the σ_0.1_ increased by more than 60% at 20 °C, while maintaining a ductility near 13%. Under tension at 800 °C, the increase in σ_0.1_ was almost 85%, but the ductility decreased to 5%. The values of σ_0.1_ after combined treatment during tension at 900 °C were 45% higher compared to TMT-II at 800 °C, while the ductility was about 9%.

Lowering the temperature of the stabilization annealing after the combined treatment to 1000 °C led to an even more significant increase in the strength properties. At 20 °C, the σ_0.1_ values were 97% higher compared to TMT-II, while the ductility level was 16%. In the case of tensioning at 800 °C, the values of σ_0.1_ were 2.3-times higher, with an acceptable level of ductility (8%).

Despite the lower volume fraction of the second phase, as a result of the lower C_Zr_ and C_O_ in the V–Cr–Ta–Zr alloy, the formation of the presented gradient state made it possible to obtain σ_0.1_ and δ at 20 °C, comparable to the V–Cr–Zr alloy properties [33] after CHT and stabilization at 1100 °C. Moreover, the V–Cr–Ta–Zr alloy yield strength was 56% and 35% higher at tensile temperatures of 800 °C and 900 °C, respectively.

**Figure 6:**
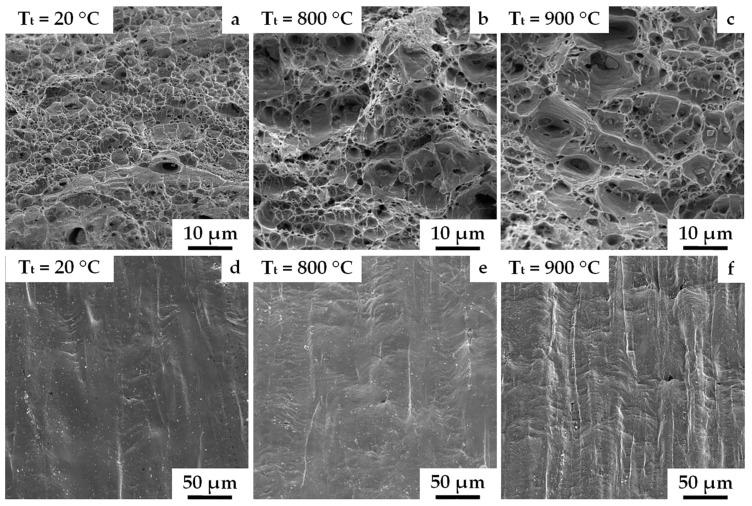
Fractographs and side surfaces of V–Cr–Ta–Zr alloy samples after combined treatment with stabilization at 1000 °C, tested at temperatures (T_t_): (**a**,**d**) T_t_ = 20 °C; (**b**,**e**) T_t_ = 800 °C; (**c**,**f**) T_t_ = 900 °C.SEM.

It was established that, regardless of the temperature of the stabilizing annealing, the V–Cr–Ta–Zr alloy was characterized by the same type of fracture at the different test temperatures. Figure 6 shows fractographs after tensioning at 20, 800, and 900 °C of specimens stabilized at 1000 °C. For all test temperatures, a viscous type of fracture was characteristic. At the same time, an increase in the tension temperature was accompanied by an increase in the cell sizes, which at 20 °C were in the range from 0.5 to 3 µm (Figure 6a), and at 800 and 900 °C were in a range from 2 to 10 µm (Figure 6b,c).

As the tension temperature increased (20, 800, and 900 °C), a developing deformation relief was formed on the side surfaces of the samples (Figure 6d–f). A slight opening of cracks along the grain boundaries in the direction perpendicular to the tension axis of the samples was observed only at 900 °C (Figure 6f). This indicated the high strength of the grain boundaries.

## 4. Discussion

As can be seen from the presented results, after combined processing, a high-defective structural-phase state was formed in the V–Cr–W–Zr alloy, which, after stabilizing annealing, was characterized by a pronounced gradient grain structure. The main reason for the formation of such a state, in our opinion, is associated with the alternation of the stages of TMT and CHT. High-defective states, as was shown in [38], provide a significant acceleration of diffusion processes. In turn, a high oxygen concentration near the oxidation surface promotes the release of a higher volume fraction of particles of the second phases. On the one hand, dispersed particles, acting as stress concentrators, contribute to a more intense fragmentation of the grain and subgrain structure in the corresponding regions at deformation stages. On the other hand, under conditions of thermal exposure, these particles contribute to the suppression of the processes of recrystallization and recovery.

Thus, the joint implementation of dispersion and substructural strengthening, in its essence, ensured the creation of a kind of structural-phase composite, in which the elements of the grain–subgrain and defect structure were fixed (stabilized) by fine particles of the non-metallic phase. At the same time, highly dispersed precipitates of the oxide phase, by pinning individual dislocations, grain boundaries, and low-angle boundaries of the substructure, prevented the recrystallization of the alloy. Moreover, using V–Cr–ZrO_2_ as an example, it was shown in [24] that, in addition to dispersion strengthening through the Orowan mechanism, fine particles of interstitial phases fix grain boundaries, thereby significantly increasing the efficiency of grain boundary strengthening, including at elevated temperatures. In addition, the high density of fine particles significantly increased the effect of substructural strengthening, which contributed to an increase in the dislocation start stress and an increase in the Hall–Petch coefficient by a factor of 1.6 compared to pure vanadium [24].

In accordance with the report in [39], the dispersion-strengthened V–Cr–Ta–Zr alloy studied in this work was characterized by a “high-strength state”, for which, regardless of the method of formation (solid-solution and dispersion strengthening, reduction of elastic moduli in alloys with structural instability, creation of submicro- and nanocrystalline structural states, change in the test temperature, high-speed deformation), under conditions of low efficiency of dislocation deformation mechanisms, plastic deformation occurred through mechanisms other than dislocation. In this case, it is important to note that, in the case of the uncorrelated motion of dislocations, the characteristic deformation volumes (volumes in which elementary acts of plastic flow occur) had dimensions in the order of the dislocation core sizes. Under conditions of low dislocation activity and the formation of high local stresses, plastic flow develops, with a significant increase in these volumes through the cooperative movement of dislocation–disclination ensembles and other high-energy carriers of crystal deformation and reorientation.

Thus, dislocation mechanisms alone are not capable of providing accommodation under conditions of active external impact. In accordance with modern concepts about the special role of grain and subgrain boundaries [40,41], during the formation of submicrocrystalline and nanocrystalline structural states in metals and alloys of different classes, it is the high length of grain boundaries that is one of the main reasons for the preservation of ductility, against the background of sometimes large increases in strength. Therefore, the formation of a polygonal state, with micron–submicron elements separated by low-angle boundaries, can provide accommodation under loading conditions, which contributes to the preservation of ductility. Depending on the external impact conditions, the contribution of the mechanisms of microstructure transformation also changes. In particular, the preservation of ductility against the background of a decrease in strength under tensile conditions at 800 °C and 900 °C is associated with the thermal activation of dislocation mechanisms, with partial degradation of their cooperative effects (ensembles, networks, walls). However, such compensation turns out to be only partial, as evidenced by the onset of crack opening along the grain boundaries on the side surfaces of the specimens under tension at 900 °C.

One of the key factors determining the thermal stability of such structural-phase composites is the thermal stability of the nanosized particles. The theoretical estimates of long-term strength (50,000 h) carried out in [42] in a quasi-binary approximation demonstrated the possibility of increasing the thermal stability of a heterophase structure and the upper limit of the operating temperature range of vanadium alloys with oxide (ZrO_2_) strengthening by 200–300 degrees compared to these alloys with the implementation of carbide (ZrC) strengthening.

The joint implementation of dispersion and substructural strengthening ensured the preservation of the polygonal structural state up to 0.6 T_melt._. As was shown in [20,43], substructural strengthening in refractory metals and their alloys is thermally stable, as a rule, up to 0.4–0.5 T_melt_.

The result of the increase in the thermal stability of the alloy was a significant increase in its strength properties, not only at 800 °C, but also at 900 °C. At the same time, an important point is the preservation of the low-temperature ductility of the alloy, which is of great importance for technological processing, both at the stage of ingot reworking and in the product manufacturing.

## 5. Conclusions

After a combined treatment, which consisted in alternating TMT and CHT cycles, the structural-phase state of the V–Cr–Ta–Zr alloy was composite, in which the joint implementation of dispersion and substructural strengthening ensured the formation of a gradient grain structure with a polygonal state, the elements of which were fixed by ZrO_2_ nanoparticles, characterized by a high thermal stability.

The formation of such a state contributed not only to an increase in the high-temperature strength, but also to a shift in the upper limit of the temperature stability interval, towards high temperatures up to 900 °C. At the same time, an increase in the high-temperature strength occurred against the background of maintaining an acceptable level of low-temperature ductility of the alloy, which is of great importance for technological processing, both at the stage of ingot processing and in the manufacture of products.

The polygonal state inside the grains, represented by fragments of micron–submicron sizes separated by low-angle boundaries, contributed to the implementation of cooperative mechanisms of the dislocation–disclination type, which provided effective accommodation of the material in the “high-strength state” under loading.

## Figures and Tables

**Table 1:** Reference data of the types and lattice parameters of ZrO_2_-type oxides.

Phase	a, Å	b, Å	c, Å	β	PDF Entry
FCC (Fm-3m)					
ZrO_1.87_	5.15	–	–	–	01-081-1551
ZrO_2_	5.14	–	–	–	01-089-9069
ZrO_2.12_	5.13	–	–	–	01-081-1550
Tetragonal (P42/nmc)					
ZrO_2_	3.67	–	5.32	–	04-005-5598
ZrO_2_	3.64	–	5.27	–	00-042-1164
ZrO_1.96_	3.62	–	5.20	–	01-081-1546
ZrO_1.99_	3.61	–	5.13	–	01-080-2155
ZrO_2_	3.60	–	5.15	–	01-088-1007
ZrO_2_	3.49	–	4.95	–	04-013-4748
Monoclinic (P21/c)					
ZrO_2_	5.31	5.21	5.15	99.22°	00-037-1484
Zr_0.93_O_2_	5.19	5.21	5.38	98.73°	01-081-1319
Zr_0.944_O_2_	5.15	5.20	5.32	99.24°	01-081-1314
ZrO_2_	5.14	5.13	5.35	98.88°	01-080-0966

**Table 2:** Yield strength (σ_0.1_) and relative elongation (δ) of the V-Cr-Ta-Zr alloy after combined treatment, depending on the stabilization annealing temperature (T_s_) and tensile temperature (T_t_).

T_s_, °C	T_t_ = 20 °C		T_t_ = 800 °C		T_t_ = 900 °C	
	σ_0.1_, MPa	δ, %	σ_0.1_, MPa	δ, %	σ_0.1_, MPa	δ, %
1000	621	16	429	8	366	8
1100	508	13	342	5	270	9

## Data Availability

Data are available on request.
